# Simvastatin Attenuates Cardiac Fibrosis via Regulation of Cardiomyocyte-Derived Exosome Secretion

**DOI:** 10.3390/jcm8060794

**Published:** 2019-06-04

**Authors:** Hsuan-Fu Kuo, Chong-Chao Hsieh, Shu-Chi Wang, Chia-Yuan Chang, Chih-Hsin Hung, Po-Lin Kuo, Yu-Ru Liu, Chia-Yang Li, Po-Len Liu

**Affiliations:** 1 Graduate Institute of Medicine, College of Medicine, Kaohsiung Medical University, Kaohsiung 807, Taiwan; medsnail@hotmail.com (H.-F.K.); kuopolin@seed.net.tw (P.-L.K.); 2 Department of Internal Medicine, Kaohsiung Municipal Ta-Tung Hospital, Kaohsiung 801, Taiwan; 3 Graduate Institute of Clinical Medicine, College of Medicine, Kaohsiung Medical University, Kaohsiung 807, Taiwan; chchhs@cc.kmu.edu.tw; 4 Division of Cardiovascular Surgery, Department of Surgery, Kaohsiung Medical University Hospital, Kaohsiung 807, Taiwan; 5 Department of Medical Laboratory Science and Biotechnology, Kaohsiung Medical University, Kaohsiung 807, Taiwan; shuchiwang@kmu.edu.tw; 6 Department of Engineering Science, National Cheng Kung University, Tainan 701, Taiwan; cychang0829@gmail.com; 7 Department of Pediatrics, Kaohsiung Medical University Hospital, Kaohsiung Medical University, Kaohsiung 807, Taiwan; pedhung@gmail.com; 8 Department of Pediatrics, Kaohsiung Municipal Hsiao-Kang Hospital, Kaohsiung 812, Taiwan; 9 Department of Respiratory Therapy, College of Medicine, Kaohsiung Medical University, Kaohsiung 807, Taiwan; lu6525@ms42.hinet.net; 10 Center for Infectious Disease and Cancer Research, Kaohsiung Medical University, Kaohsiung 807, Taiwan; 11 Department of Medical Research, Kaohsiung Medical University Hospital, Kaohsiung 807, Taiwan; 12 Regenerative Medicine and Cell Therapy Research Center, Kaohsiung Medical University, Kaohsiung 807, Taiwan

**Keywords:** statins, exosomes, decorin, periostin, cardiac fibrosis

## Abstract

Exosome-mediated communication within the cardiac microenvironment is associated with cardiac fibrosis. Simvastatin (SIM), a potent statin, protects against cardiac fibrosis, but its mechanism of action is unclear. We investigated the inhibitory effects and underlying mechanism of simvastatin in cardiac fibrosis, by regulating exosome-mediated communication. Male Sprague-Dawley rats were treated with angiotensin (Ang) II alone, or with SIM for 28 d. Cardiac fibrosis, expressions of fibrosis-associated proteins and mRNAs, and collagen fiber arrangement and deposition were examined. Protein expressions in exosomes isolated from Ang II-treated cardiomyocytes (CMs) were evaluated using nano-ultra-performance liquid chromatographic system, combined with tandem mass spectrometry. Transformation of fibroblasts to myofibroblasts was evaluated using scanning electron and confocal microscopy, and migration assays. Our results showed that SIM attenuated in vivo expression of collagen and collagen-associated protein, as well as collagen deposition, and cardiac fibrosis. The statin also upregulated decorin and downregulated periostin in CM-derived exosomes. Furthermore, it suppressed Ang II-induced transformation of fibroblast to myofibroblast, as well as fibroblast migration. Exosome-mediated cell-cell communication within the cardiac tissue critically regulated cardiac fibrosis. Specifically, SIM regulated the release of CM exosomes, and attenuated Ang II-induced cardiac fibrosis, highlighting its potential as a novel therapy for cardiac fibrosis.

## 1. Introduction

Cardiac fibrosis is a common feature of progressive coronary heart disease, and is associated with hypertension, myocardial infarction, cardiomyopathy, and heart failure. Angiotensin II (Ang II)-induced pressure overload is a common model of cardiac fibrosis progression [1,2]. A previous study confirmed that cardiac fibrosis played a key role in the progression of heart failure [3]. However, no clinically effective therapies have been developed to prevent, treat, or even reverse the progression of cardiac fibrosis.

The heart consists of cardiomyocytes (CMs), and non-CMs, including fibroblasts, and endothelial, smooth muscle, stem, and immune cells [4]. CMs and fibroblasts are the most abundant and intermingled cells in the heart. Communication between myocardial cells and non-CMs plays a key role in maintaining a homeostatic microenvironment and regulating disease progression [5]. Extracellular vesicles (exosomes and microvesicles) are released from most cells as critical messengers in cardiac injury, repair, and remodeling [6]. Previous studies have revealed that transport of extracellular vesicles between cardiovascular progenitor cells and CMs contributes to improved cardiac function, and attenuates heart failure progression [7]. Exosomes enriched in miR-21 from cardiac fibroblast (CF) act as paracrine signaling mediators to regulate CM hypertrophy [8]. Mesenchymal stem cell-derived exosomes stimulated CM proliferation and inhibited apoptosis, and aid in transformation of fibroblast to myofibroblast, after induction with transforming growth factor (TGF)-β to enhance cardiac repair [9]. Therefore, exosome-mediated cardiac cell communication represents a potential future target for treating or preventing a variety of cardiac diseases, including cardiac fibrosis [10].

Excessive deposition of extracellular matrix in the myocardium is an important feature of cardiac fibrosis. Additionally, transformation of CF to myofibroblast enhanced CF migration, collagen production, matrix deposition, and extracellular protein expression during the progression of cardiac fibrosis [11]. Previous studies have demonstrated that microRNA-enriched exosomes derived from mesenchymal stem cells suppressed myofibroblast formation during skin wound healing, by inhibiting α-smooth muscle actin (α-SMA) expression, collagen deposition, and extracellular matrix remodeling [12]. Other studies also indicated that, after cigarette smoke exposure, bronchial epithelial cell-derived exosomes could promote fibroblast to myofibroblast differentiation in airway remodeling, which contributed to chronic obstructive pulmonary disease [13]. Nevertheless, whether CM-derived exosomes act as regulators of myofibroblast differentiation in cardiac fibrosis is unknown.

Collagens accumulate in the interstitium of the heart, playing a key role in the progression of cardiac fibrosis [14]. COL1, the major component of the extracellular matrix, is encoded by *COL1A1* and COL1A2 genes. In addition, the regulation of COL1A1 transcription has been widely studied in the progression of pathological fibrosis [15]. Lysyl oxidase like-2 (LOXL2) belongs to the LOX family and mediates collagen stabilization, deposition, and induction of α-SMA in fibroblasts to promote fibrosis progression [16]. Furthermore, Ang II-dependent cardiac fibrosis promotes collagen synthesis, deposition, and matrix remodeling through the up-regulation of COL1A1 and LOXL2 expressions in the myocardium [17].

Simvastatin (SIM) is a potent competitive inhibitor of 5-hydroxy-3-methylglutaryl-coenzyme A reductase, and has cardiovascular protective effects, which prevents cardiovascular-related mortality and morbidity [18]. Another statin, Atorvastatin attenuates Ang II-induced cardiac fibrosis by down-regulating the TGF-β1/Smad2/3 activation pathway, and up-regulating TGF-β receptor III expression in CF [19]. However, numerous questions regarding the role of statins and SIM in the heart remain and require further investigation.

Therefore, the objective of this study was to investigate whether the regulation of exosomes by SIM in the myocardial microenvironment participated in cardiac fibrosis. To this purpose, we established an animal model of cardiac fibrosis, and human cardiomyocyte (HCM) and human cardiac fibroblast (HCF) cell lines to explore the involvement of exosomes in cardiac fibrosis.

## 2. Experimental Section

### 2.1. Animals and Treatment

The animal procedures were conducted in strict compliance with Taiwan legislation. All animal experiments were approved by the Institutional Animal Care Committee of Kaohsiung Medical University (Permit Number: 106039) and performed in an Association for Assessment and Accreditation of Laboratory Animal Care International (AAALAC)-accredited facility. The 7-week-old male Sprague-Dawley rats were purchased from BioLASCO Taiwan Co., Ltd. (Taipei, Taiwan). All rats were weighed weekly during the experimental period. There were three experimental groups (each group *n* = 6: (1) PBS (vehicle); (2) Ang II (1 mg/kg/day) (Merck, Kenilworth, NJ, USA) via subcutaneously implanted Alzert osmotic pump (infusion rate 0.5 μL/h) over 28 days; (3) Ang II + simvastatin (oral, 10 mg/kg, Merck, Kenilworth, NJ, USA). After 28 days, the rats were euthanized with CO_2_ and their hearts were immediately harvested for further analyses. In the experiment, if the body weight was excessively reduced (≥20% of the initial body weight), the rats were to be necessarily excluded from the experiment.

### 2.2. Immunostaining and Masson’s Trichrome Staining

For assessment of cardiac fibrosis morphological changes, 5 μm thick tissue sections of left ventricle tissue were analyzed by Masson’s trichrome staining kit (Invitrogen, Carlsbad, CA, USA). Fibrosis area (blue color) and whole section areas were analyzed using NIH ImageJ (Bethesda, MD, USA), following a procedure as described previously [20]. To quantify the fibrosis-related protein expressions, tissue sections or cultured cells were incubated in blocking buffer (0.5% bovine serum albumin, BSA, 0.05% Tween-20, and PBS) for 1 h, followed by specific primary antibodies: COL1A1 (Gene Tex, Irvine, CA, USA), LOXL2 (Gene Tex, Irvine, CA, USA), α-SMA (Abcam, Cambridge, MA, USA) and F-actin (Invitrogen, Carlsbad, CA, USA) for 1 h. The antibody staining was developed using a fluorescence detection system (Ventana Medical Systems, Invitrogen, Carlsbad, CA, USA) for 1 h, and counterstained with DAPI (4′,6-diamidino-2-phenylindole, Invitrogen, Carlsbad, CA, USA). After washing, sections were mounted in VECTASHIELD^®^ mounting medium (Invitrogen, Carlsbad, CA, USA) and examined under a confocal laser miscopy (Leica, Baca Raton, FL, USA). 

### 2.3. Transmission Electron Microscopy

Transmission electron microscopy (TEM) analyses were performed as described previously [21]. In brief; tissue samples were fixed with 2.5% glutaraldehyde for 2 h at 4 °C. After washing, the samples were post-fixed in 1% osmium tetroxide for 2 h, dehydrated in graded acetone, infiltrated, and then embedded in Epoxy resin. Ultrathin 70 nm sections were cut using a Leica microtome (Leica RM2165, Tokyo, Japan) and examined using TEM (HITACHI HT-7700, Tokyo, Japan) at an accelerating voltage of 80 kV.

### 2.4. Scanning Electron Microscopy

Scanning electron microscopy (SEM) analyses were performed as described previously [22]. The cultured cells were seeded on coverslip (0.17 mm thickness) and fixed in 2.5% glutaraldehyde overnight at 4 °C. After washing, the samples were post-fixed in 2% osmium tetraoxide (OsO4) for 1.5 h at 4 °C, and dehydrated in ascending grades of alcohol (50%, 75%, 85%, 95% and 100%). The cells were dried by the critical point drier (CPD 030, Bal-TEC, Heerbrugg, Switzerland) for 1 h, and then coated in gold and monitored under field emission scanning electron microscopy (Hitachi-8010, Tokyo, Japan) at accelerating voltage of 10–25 KV.

### 2.5. Second-Harmonic Generation Microscopy

Collagen deposition in cardiac sections was analyzed by Second-harmonic generation microscopy (SHG). Multiphoton imaging was performed using 10 μm thick acute slices without de-waxing and staining, and acquisitions were taken with a high numerical aperture (NA) objective lens (UPlanSAPo 20×/NA 0.75, Olympus, Tokyo, Japan) which enabled visualization of a major part of the slice. Visualization of micrometric or submicrometric collagen fibers that were heterogeneously distributed in the cardiac tissue at millimeter scale required both a large field of view and a good spatial resolution. The SHG signal was excited by a femtosecond Ti:Sapphire laser (Tsunami, Spectra-Physics, Mountain View, CA, USA) adjusted to wavelength of 800 nm and a fluorescence filter (FF01-390/40-25, Semrock, Rochester, NY, USA) specifically chosen for SHG detection. We therefore acquired 200 × 200 μm^2^ SHG images with 512 × 512 pixels at a pixel scanning rate of 30 kHz and used laser power of 22.4 mW on the specimen. Data analysis (threshold adjustment and SHG scoring) was routinely performed using macros established under ImageJ free software and UCSD plugins respectively.

### 2.6. Cell Culture

HCM (ScienCell Research Laboratories, Carlsbad, CA, USA) and HCF (C-12375, PromoCell, Heidelberg, Germany) cell lines were cultured in a cardiac myocyte medium (ScienCell Research Laboratories, Carlsbad, CA, USA), and HCF growth medium (C-23010, PromoCell, Heidelberg, Germany) supplemented with 5% fetal bovine serum (FBS), 1% cardiac myocyte growth supplement (ScienCell Research Laboratories, Carlsbad, CA, USA), and 1% penicillin/streptomycin solution (ScienCell Research Laboratories, Carlsbad, CA, USA). Cells were cultured in an atmosphere of 5% CO_2_ at 37 °C, and the incubation medium was replaced every 3–4 days.

### 2.7. Exosomes Isolation and CM-DiI Labelling

Human cardiomyocyte cells (5 × 10^6^) were cultured until 90% confluence and the culture medium (FBS free) was harvested. The method of cardiomyocyte exosome isolation was according to the manufacturer’s instructions (ThermoFisher scientific exosome isolation kit, catalog # 4478359). Cell culture supernatant was centrifuged by different centrifugation steps: 400× *g* for 10 min; 2000× *g* for 30 min and 15,000× *g* for 30 min were performed to remove leftover cells, cellular debris or microparticles. The size of exosomes was examined by NanoSight NS300 (NanoSight Ltd., Amesbury, UK) nanoparticle tracking analysis and SEM, and the expression of exosomal markers was detected by Western blot (a total of 40 μg protein in each group). For the DiI labeling, the isolated exosomes (5 μg/mL) were incubated with CM-DiI (CellTracker^TM^ CM-DiI; C7001, Carlabad CA, USA) at 37 °C for 1 h, and then CM-DiI labeled exosomes were washed twice by PBS before incubation with fibroblast.

### 2.8. Western Blotting

Protein concentrations of cell, exosome and tissue lysates were measured using the Lowry assay. A total of 20 µg proteins were separated by SDS-PAGE in 12% gels depending on the expected molecular weight of the target. Proteins were transferred onto nitrocellulose membranes, which were blocked with 5% non-fat dry milk, and incubated with primary antibodies against CD81 (1:500; GTX101766, Gene Tex, Irvine, CA, USA), CD63 (1:500, NBP2-42225SS, Novus Biologicals, Littleton, CO, USA), HSP70 (1:1000, MA-006, ABR Affinity BioReagents, Golden, CO, USA), Calnexin (1:500, GTX109669, GeneTex), β-actin (1:2000; GTX109639. Gene Tex), and GAPDH (1:2000, sc-137179, Santa Cruz Biotechnology, Santa Cruz, CA, USA). Incubation with horseradish peroxidase-conjugated secondary antibodies was then performed, and the signals were then visualized using an enhanced chemiluminescence detection kit (TOOLS Extreme ECL-HRP Substrate, New Taipei City, Taiwan).

### 2.9. Real-Time (RT)-PCR

Total RNA was extracted from tissues or cells using RNA purification kits (Invitrogen, Carlsbad, CA, USA). cDNA was synthesized from 2 μg total RNA using SuperScriptTM and First-Strand Synthesis System for RT-PCR kit (Invitrogen, Carlsbad, CA, USA). RNA expression levels were determined by real-time PCR using SYBR Green PCR (LightCycler FastStart DNA Master SYBR Green I, Roche) and target gene-specific for rat *LOXL2* (5′-TGA CTG CCA GTG GAT AGA C-3′ and 5′-ATG CGG TAG CCA TCA TAG C-3′); human *LOXL2* (5′-GCG TCA CTG ACT GCA AGC AC-3′ and 5′-CGA ATC CGA ATG TCC TCC AC-3′); rat *COL1A1* (5′-ACA GCG TAG CCT ACA TGG-3′ and 5′-AAG TTC CGG TGT GAC TCG-3′); human *COL1A1* (5′-GGA CAC AGA GGT TTC AGT GGT-3′ and 5′-CAC CAT CAT TTC CAC GAG CA-3′); rat *ACTA2* (5′-ACC TTC AAT GTC CCT GCC ATG TA-3′ and 5-ACG AAG GAA TAG CCA CGC TCA-3′); human *ACTA2* (5-ACT GCC TTG GTG TGT GAC AAT GG-3′ and 5-TGG TGC CAG ATC TTT TCC ATG 3′); rat *β-actin* (5′-TGT CAC CAA CTG GGA CGA TA-3′ and 5′-TCT CAG CTG TGG TGT GAA G-3′), and human *β-actin* (5′-GCC GCC AGC TCA CCA T-3′ and 5′-TCG ATG GGG TAC TTC AGG GT-3′). PCR conditions included an initial denaturation at 94 °C for 180 s, followed by 40 cycles at 95 °C for 30 s, 60 °C for 25 s, 72 °C for 30 s, and 1 cycle at 72 °C for 7 min. Fluorescence data were acquired after the final extension step. A melt analysis was conducted for all products to determine the specificity of the amplification. Relative mRNA levels were normalized by housekeeping gene *β*-actin.

### 2.10. Cell Migration and Motility Analysis

IBIDI™ Culture Inserts (IBIDI, Madison, WI, USA) were placed into 6-well culture dishes and 1 × 10^4^ cells/mL HCF cells were seeded into the two reservoirs of the same insert. There were three experimental groups: (1) Exosome; (2) Ang II-exosomes (pretreated with Ang II for 24 h); and (3) Ang II-SIM-exosomes (pretreated with simvastatin and Ang II for 24 h). After 24 h treatment, the insert was removed with caution, creating a gap of 0.5 mm, and cell migration was followed by bright-field microscopy at specific time points (0 h and 24 h). The migrating HCF cells were photographed, and the cell-covered areas were measured and quantified by the Wimasis WimScratch technique. HCF motion ability, tract, displacement, and velocity were measured by time-lapse confocal microscopy (SP2; Leica, Exton, PA, USA) at various time points (0–20 h).

### 2.11. Nano Ultra-performance Liquid Chromatographic System with Tandem Mass Spectrometry

Nano ultra-performance liquid chromatographic system with tandem mass spectrometry (nanoUPLC-MS/MS) analyses were performed as described previously [12]. The nanoUPLC was purchased from Waters (Milford, MA, USA) and MS/MS was performed with an LTQ Qrbitrap Discovery hybrid Fourier Transform Mass Spectrometer (Thermo Fisher Scientific, Inc. Bremen, Germany). Extracellular exosomes were isolated and then analyzed by nanoUPLC-MS/MS. Protein solution (10 μg in 10 μL) was mixed well with 100 μL acetone and centrifuged at 10,000 g for 10 min. After centrifugation, the supernatant was discarded, and the protein residues were preserved and evaporated to dryness. Protein residues were re-dissolved with 16 μL ammonium bicarbonate aqueous solution (25 mM). After trypsin digestion, 2 µL of tryptic peptide solution was injected into the nanoUPLC system and detected by LTQ Orbitrap. The peptide eluate from the column was directed to the nanospray source, and the MS was operated in positive ion mode. The MS/MS were acquired with a mass spectrometer operated in data-dependent mode. Individual raw data files were processed with Mascot Distiller software and then uploaded to the in-house Mascot server or protein identification.

### 2.12. Statistical Analysis

The data are presented as the means ± standard error of the mean (SEM) of each group and were analyzed using ANOVA followed by Dunnett’s test. All statistics were calculated using the SigmaStat version 3.5 (Systat Software Inc., Chicago, IL, USA), and a *p* < 0.05 or *p* < 0.01 was considered statistically significant.

## 3. Results

### 3.1. SIM Suppresses Ang II-Mediated Collagen Deposition and Cardiac Fibrosis In Vivo

**Figure 1:**
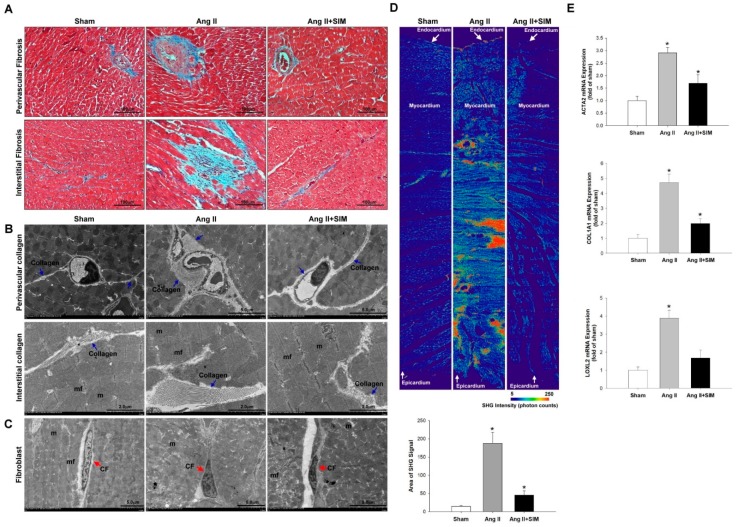
Simvastatin suppresses in vivo angiotensin (Ang) II-mediated collagen deposition, fibrosis, and collagen-associated protein expression. Male Sprague-Dawley rats were treated with (Ang II, 1 mg/kg/day) or Ang II + simvastatin (SIM, oral, 10 mg/kg) for 28 days. Perivascular and interstitial fibrosis ultrastructures (blue color) were determined using Masson’s trichrome staining. (**A**) The collagen fibers are shown in the representative images (blue) (*n* = 6). Perivascular and interstitial collagen fiber expression (blue arrows) (**B**) and quantification by transmission electron microscopy (TEM) analysis are shown (Supplemental Figure S1A,B). The cardiac fibroblast (CF) ultrastructures (red arrows) were determined using TEM analysis (**C**) (*n* = 6) (mf, myofibril; m, mitochondrial; CF, cardiac fibroblast). Cardiac collagen deposition and arrangement from endocardium to epicardium were identified and quantified using second-harmonic generation (SHG) microscopy (**D**) (*n* = 3). * *p* < 0.05 vs. the control from three independent experiments. The fibrotic associated genes: *ACTA2*, *COL1A1*, and *LOXL2* expression were analyzed using real-time PCR and normalized by β–actin (**E**). For all comparisons, * *p* < 0.05 vs. sham.

Masson’s trichrome staining indicated that Ang II markedly increased perivascular and interstitial cardiac fibrosis (collagen, blue color), compared with that of the sham or (Ang II + SIM) group (Figure 1A). However, the induction of fibrosis was significantly inhibited by SIM treatment. To determine the effects of SIM on collagen deposition in Ang II-derived cardiac fibrosis, TEM and SHG microscopy (endocardium to pericardium) analyses were used and quantified (supplement Figure S1). The results showed that Ang II increased collagen deposition in perivascular and interstitial (collagen, blue arrow) (Figure 1B) and massive collagen accumulation in myocardium (Figure 1D). However, SIM treatment significantly inhibited Ang II-mediated collagen deposition (Figure 1D).

Fibroblasts are non-CM cells that play a key role in maintaining a homeostatic microenvironment, and collagen generation. Proto-myofibroblasts further develop into ‘differentiated myofibroblasts’, based on the high expression of myofibroblast marker ACTA2 [23]. Collagen and the collagen-associated genes, *COL1A1* and *LOXL2*, are essential for the biosynthesis, and stabilization of collagen respectively [24]. To determine whether the Ang II affected fibroblast to myofibroblast transition, TEM analysis was performed (Figure 1C). The experimental results showed that fibroblasts appeared as elongated cells with thin flat/wavy nuclei (inactive fibroblasts) in sham and Ang II+SIM groups. However, Ang II-induced myofibroblasts (inactive fibroblasts) are characterized by large, spindle-shaped stellate cells with long cytoplasmic extensions and nuclei with conspicuous nucleoli. These results indicated that SIM inhibited Ang II-induced fibroblast to myofibroblast transition. Specifically, SIM significantly suppressed the mRNA expressions of fibrotic associated genes *ATCA2*, *COL1A1*, and *LOXL2* in cardiac tissue (Figure 1E), as observed by real-time PCR.

### 3.2. Ultrastructure between Cardiomyocyte and Fibroblast of the Heart In Vivo

**Figure 2:**
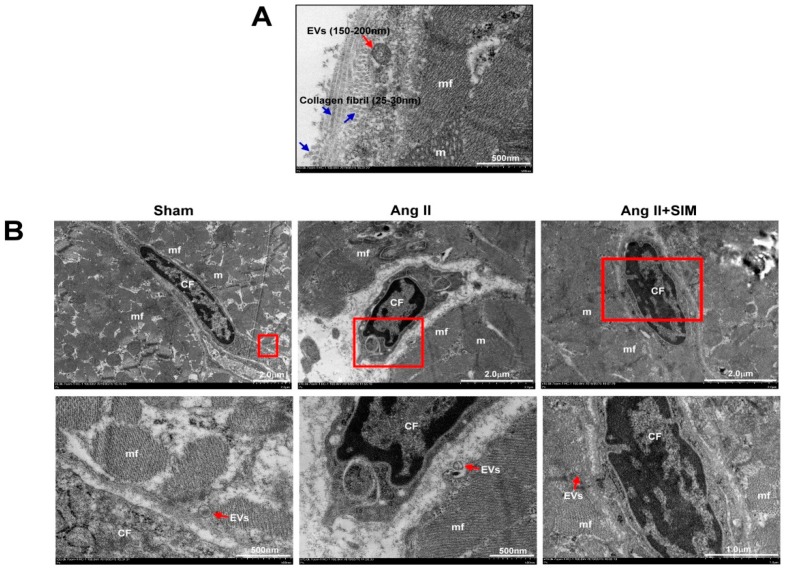
Ultrastructure between CM and CF of the heart in vivo. The SD rats were divided into sham group, Ang II group and Ang II + SIM group (each group *n* = 6). TEM images of rat left ventricles demonstrated the morphology, size of longitudinal and cross-sectional collagen fibers, and extracellular vesicles (EVs) (**A**). The distribution of EVs between CM and CF was explored by TEM analysis (**B**) (mf, myofibril; m, mitochondrial; CF, cardiac fibroblast).

In cardiac fibrosis, CF acts as key cells in scaffold production, collagen deposition, extracellular matrix construction, and tissue stabilization [25]. In order to direct intercellular communications between cardiomyocyte (CM) and neighboring CF, CM-secreted extracellular vesicles (EVs) containing microvesicles (size 100–1000 nm) and exosomes (size 30–100 nm) might be delivered by paracrine to CF [26,27]. The size and expression of collagen fibrils and EV-like structures were examined by TEM. The experimental results revealed that the EV-like structures were fused with the CM plasma membrane, and collagen fibers were accumulated in extracellular space (Figure 2A). In addition, we also explored the crosstalk between CM and CF by TEM. As shown in Figure 2B, CM generated and secreted MV-like structures into the CM-CF intercellular junctions for each group.

### 3.3. HCM-Secreted Exosomes Are Transported to HCFs In Vitro

**Figure 3:**
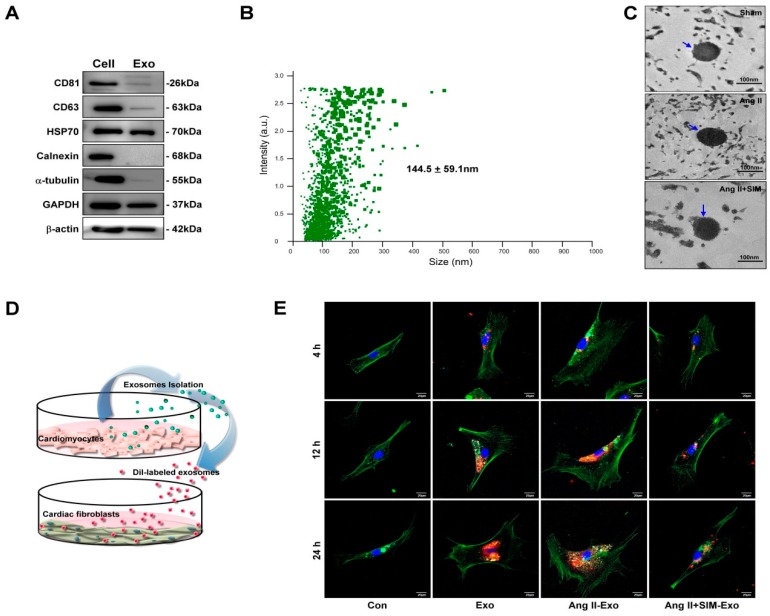
HCM-derived exosomes are transported into human cardiac fibroblasts (HCF) in vitro. Exosomal-specific marker expression from HCM-isolated exosomes was measured using Western blot analysis (**A**). HCM-isolated exosome size was demonstrated by NanoSight NS300 (**B**) and TEM analysis (**C**). To identify uptake of HCM-derived exosomes by HCF, exosomes were isolated from HCM-conditioned medium, after treatment with Ang II, or Ang II + SIM for 24 h. HCM exosomes were labeled with the DiI dye (5 μg/mL, red florescence) for 1 h, and incubated with HCF cells for various times (4, 12, and 24 h) (**D**). HCF cells were non-treated (control), or treated with Eox, Ang II-Exo and Ang II-SIM-Exo for various times (4, 12, and 24 h), then stained for intracellular cytoskeleton F-actin (green florescence; nucleus, blue florescence), and analyzed by confocal microscopy (**E**). Scale bars: 20 μm. Each image is representative of three independent experiments.

To identify the HCM-secreted EVs containing exosomes, the HCM-cultured medium was collected and exosomes were extracted. Exosome-specific markers, CD81, CD63 and HSP70, and an endoplasmic reticulum marker, calnexin, were examined by Western blot. As shown in Figure 3A, exosome-specific markers, CD81, CD63 and HSP70, were detected in exosomal extracts, but not calnexin. Furthermore, the size of the isolated exosome was measured by NanoSight NS300 nanoparticle tracking analysis and TEM. Results of nanoparticle tracking analysis and TEM showed that the size distribution of exosome samples was 144.5 ± 59.1 nm and 80–120 nm (Figure 3C) respectively. These results showed that HCM-derived exosomes were successfully isolated, and there was no difference in exosome size between groups. To identify CM-derived exosomes transported into neighboring HCFs, exosomes were labeled with a fluorescent DiI dye for 1 h, and then incubated with HCF cell line at different time points: 4, 12, and 24 h (Figure 3D). The uptake of exosomes by fibroblasts was analyzed using confocal microscopy. Our experimental results indicated that the accumulation of exosomes in the Ang II group was higher than in other groups. However, SIM pre-treatment effectively reduced the accumulation of exosomes in fibroblasts (Figure 3E). Our findings demonstrated that CM-derived exosomes mediated paracrine signaling in the crosstalk between HCM and HCF cells, and SIM reduced exosomes uptake by HCF cells.

### 3.4. SIM Suppresses Fibroblast to Myofibroblast Transformation, and Fibrosis-Associated Protein Expression In Vitro

**Figure 4:**
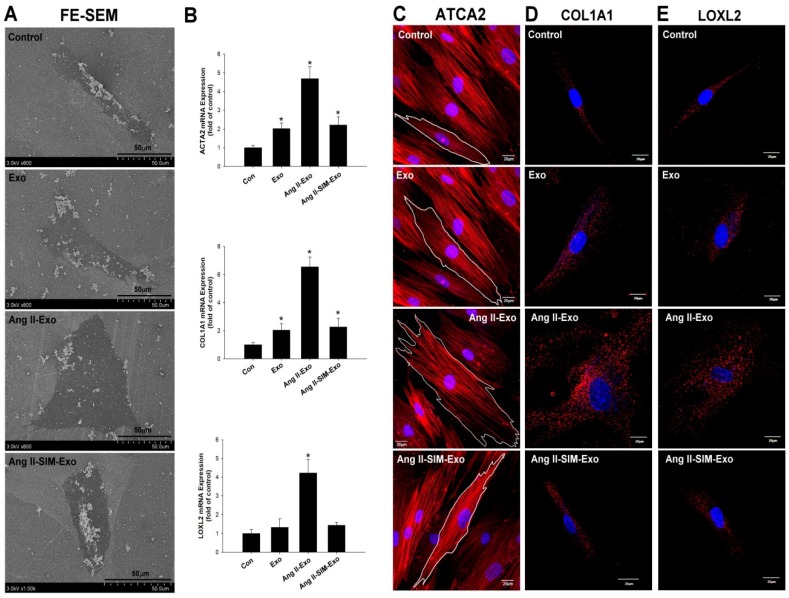
Simvastatin attenuates myofibroblast phenotype transformation and collagen-associated protein expression in vitro. Exosomes were isolated from HCM culture medium pretreated with Ang II or Ang II + SIM for 24 h. Exosomes were then incubated with HCF for 24 h. HCF ultrastructure images were examined using scanning electron microscopy (SEM) (**A**). mRNA expressions of myofibroblast markers ATCA2, COL1A1, and LOXL2 were examined by real-time PCR and normalized by β–actin (**B**). Data represent mean ± SD of three independent experiments (* *p* < 0.05). Confocal microscopy was used to confirm ATCA2, COL1A1, and LOXL2 expressions (red florescence: ATCA2, COL1A1, LOXL2; blue florescence, the nuclei) (**C–E**). A representative image from three independent experiments.

To explore Ang II-increase of CM-derived exosomes, which mediate fibroblast to myofibroblast transformation, ultrastructural images of HCF cells were acquired using SEM analysis. Our experimental results showed that inactive fibroblasts (called fibrocytes) were smaller, thin, and spindle-shaped in the control group (Figure 4A). After HCF cells were treated with HCM-derived exosomes for 24 h, the exo and Ang II+SIM groups showed a plump spindle shape compared to the control group (Figure 4A). Different from other groups, Ang II-Exo group appeared as plump spindle-shaped or large stellate-shaped cells (active fibroblasts) (Figure 4A). The expression of myofibroblast markers, ACTA2, COL1A1, and LOXL2, was measured using real-time PCR, and immunofluorescence staining. When fibroblasts transformed into myofibroblasts, their expression of fibrosis-associated proteins e.g., increased to promote fibrosis [28]. Our results indicated that Ang II-Exo promoted fibroblast to myofibroblast transition, by increasing HCF cell size, surface area, and expression of *ATCA2*, *COL1A1*, and *LOXL2* genes (Figure 4B). In addition, co-administration of SIM with Ang II Exo remarkably suppressed this transformation (Figure 4C–E).

### 3.5. SIM Suppresses HCF Migration Induced by CM-Derived Exosomes In Vitro

**Figure 5:**
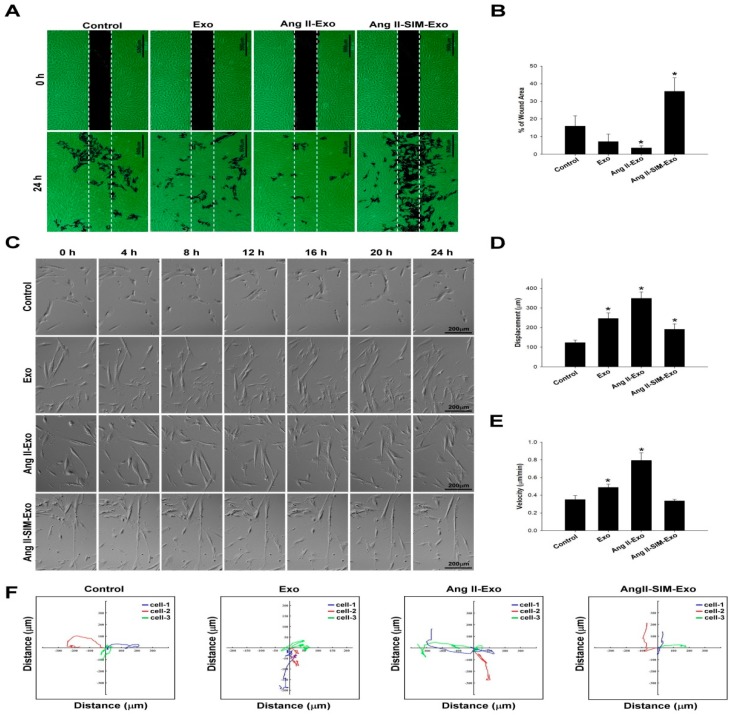
Simvastatin inhibits HCF migration after induction with HCM-derived exosomes in vitro. Exosomes were isolated from the culture medium of HCM cells pretreated with Ang II or Ang II + SIM for 24 h, and then HCF cells were incubated with human HCF cells for 24 h. HCF cell migration mediated by HCM-derived exosomes was analyzed (**A**), and quantified (**B**) by wound healing assay. Scale bar, 500 µm. * *p* < 0.05 vs. control from three independent experiments. To reveal simvastatin inhibition of HCF cell mobility, time-lapse confocal microscopy was used. Images were acquired (**C**), cell movements were measured, and displacement (μm) (**D**), velocity (μm/min) (**E**), and motility tracks (x and y axis; distance μm) (**F**) were quantified. * *p* < 0.05 vs. control from three independent experiments.

Ang II stimulates HCF migration by up-regulating collagen generation and matrix metalloproteinase (MMP) activity [29]. To explore the anti-migratory effects of SIM on CM-derived exosome-mediated HCF migration, we first evaluated HCF cell migration using a wound healing assay (Figure 5A,B). Additionally, we examined HCF cell migration images (Figure 5C) to determine migration displacement (μm) (Figure 5D), and velocity (μm/min) (Figure 5E), and we tracked the motility using time-lapse confocal microscopy (x and y axis; distance μm) (Figure 5F). Similar effects were observed in both assays; specifically, co-administration of SIM significantly attenuated CM-derived exosome-mediated HCF cell migration and motility.

### 3.6. SIM Regulates HCM-Derived Exosome Protein Expression In Vitro

**Figure 6:**
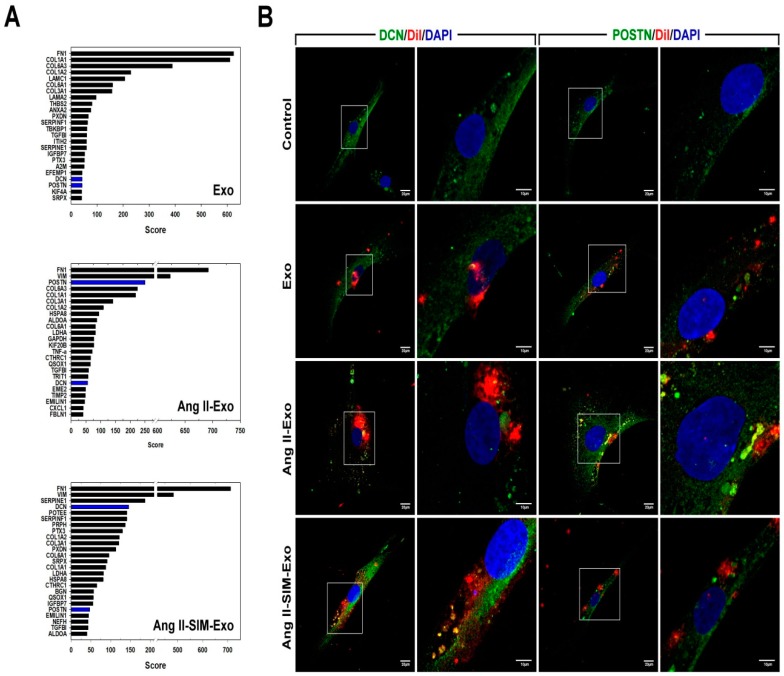
Simvastatin regulates HCM-derived exosome protein content in vitro. HCM-derived exosome proteins were isolated after Ang II or Ang II+SIM treatment and identified using nano UPLC-MS/MS (**A**). To confirm exosomal decorin (DCN) and periostin (POSTN) protein expressions, HCM-derived exosomes were labeled with DiI dye (5 μg/mL, red florescence) for 1 h, and then incubated with HCF cells for 24 h. Immunofluorescence staining was performed (DCN and POSTN, green fluorescence; exosomes-labeled with DiI dye, red fluorescence; nucleus, blue fluorescence) in HCF cells (**B**). A representative image from three independent experiments is shown.

Exosomes contain a variety of biological signal transmitters, including miRNAs and proteins, which are crucial for exosome-mediated cell communication [30]. To study the protein content of HCM-derived exosomes from primary cultures, we isolated HCM-derived exosomes from conditioned medium after treatment with Ang II alone, or co-administered with SIM for 24 h. Nano UPLC-MS/MS results showed that Ang II treatment upregulated periostin (POSTN) expression, compared with that of the control or Ang II + SIM group (Figure 6A). POSTN is a pro-fibrotic protein that plays a key role in extracellular matrix deposition, mesenchymal cell proliferation, wound closure, and fibrosis [31,32]. In addition to suppressing POSTN expression in HCM-derived exosomes, SIM significantly enhanced decorin (DCN) expression. DCN is an extracellular matrix protein that regulates a wide range of biological processes, including cell growth, differentiation, proliferation, adhesion, migration, and fibrosis [33,34]. We next confirmed POSTN and DCN expressions in isolated exosomes using immunocytochemical staining after HCF was treated by Dil-labeled exosome for 24 h (Figure 6B). Collectively, these data showed that SIM modulated the exosomal expressions of DCN in exosomes (DCN, green florescence; exosome, red florescence; nuclear, blue florescence) and POSTN (POSTN, green florescence; exosome, red florescence; nuclear, blue florescence) by upregulating and downregulating their expressions respectively, to suppress HCF-mediated collagen production and cell motility.

### 3.7. DCN Inhibits Collagen-Associated Gene Expression, Fibroblast Transformation, and Cell Motility In Vitro

**Figure 7:**
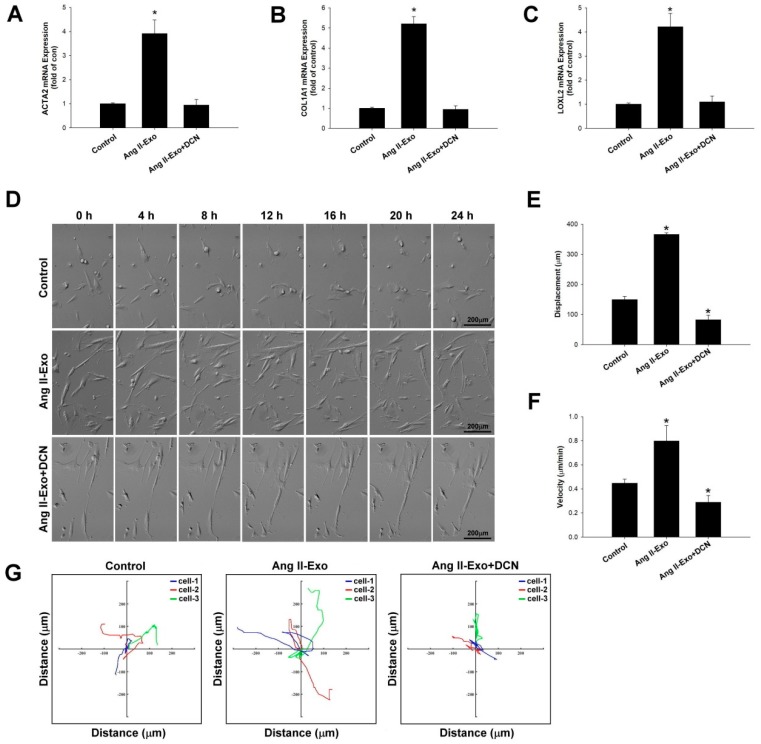
DCN inhibits collagen-associated gene expression, fibroblast transformation, and cell motility in vitro. The effects of DCN on *ATCA2*, COL1A1, and LOXL2 expressions were examined using real-time PCR and normalized by β–actin (**A**–**C**). * *p* < 0.05 vs. control from three independent experiments. DCN effect on HCF cell migration was examined using time-lapse confocal microscopy. Images were acquired (**D**), and cell movements measured as displacement (μm) (**E**), velocity (μm/min) (**F**), and motility tracks (x and y axis; distance μm) (**G**), were plotted. * *p* < 0.05 vs. control from three independent experiments.

To verify the role of DCN in HCF transformation mediated by HCM-derived exosomes, collagen-associated gene expression of *ATCA2*, *COL1A1*, and *LOXL2* was measured using real-time PCR (Figure 7A–C). Furthermore, HCF cell migration images (Figure 7D) were acquired, and migration displacement (μm), velocity (μm/min), and motility tracks (x and y axis; distance μm) (Figure 7E–G respectively) were examined using time-lapse confocal microscopy. Our data showed that DCN significantly suppressed fibroblast transformation, ATCA2, COL1A1, and LOXL2 expressions, and fibroblast motility of Ang II-induced HCM-derived exosomes.

## 4. Discussion

Statins (for example, SIM, atorvastatin, rosuvastatin, etc.) have been demonstrated to possess various biological functions, not only in lowering cholesterol levels but also inhibiting inflammatory response [35], preventing contrast-induced acute kidney injury (CIAKI) [36], and reducing cardiovascular risk [37] and anti-fibrosis effect [38]. SIM is the earliest statin, and is commonly used for hyperlipidemia, and is easy to obtain. Xinwei et al. indicated that high-dose SIM (80 mg) before percutaneous coronary intervention decreases the occurrence of CIAKI compared to low-dose SIM (20 mg) [39].

Exosomes are intracellular and cell-cell communication mediators, involved in the development of cardiac disease [16,40,41,42]. A previous study demonstrated that Ang II-mediated exosome secretion from HCF regulated heart failure progression via up-regulation of the renin-angiotensin system [43]. Similar studies also indicated that HCF produced and released miR-21-enriched exosomes, which were taken up by CM, thus leading to cardiac hypertrophy and fibrosis [44]. HCF activation and differentiation into myofibroblasts promote cardiac fibrosis progression; however, the underlying mechanisms of CM-fibroblast communication in cardiac fibrosis and myofibroblast phenotypic expression that are regulated by exosomes remain unclear. Several studies demonstrated that SIM had an anti-fibrosis effect, such as reducing TGF-β1-induced SMAD2/3-dependent pathway activation and promoted human ventricular fibroblasts differentiation [45]. It also alleviated cardiac fibrosis-induced infarction by up-regulating TGF-β receptor III expression [25]. Finally, SIM was shown to attenuate the oxidative stress, endothelial thrombogenicity, and the inducibility of atrial fibrillation in a rat model of ischemic heart failure [46]. However, the effect of SIM on exosome communication within the fibrotic microenvironmental has not been examined. We hypothesized that SIM inhibits Ang II-induced cardiac fibrosis by exosome regulation.

We investigated a potential paracrine protein crosstalk between CM and CF. Notably, Ang II markedly up-regulated POSTN expression in CM-derived exosomes. Conversely, SIM treatment significantly down-regulated POSTN expression, but up-regulated DCN expression in Ang II-induced CM-derived exosomes. POSTN is a 93-kDa secreted matricellular protein, which has been implicated in cell adhesion and migration, cell-matrix interactions, wound healing, cardiac remodeling, and cardiac fibrosis [33]. Previous studies indicated that POSTN is abundant in embryonic cardiac tissue [47], diabetic cardiomyopathy [48], the aging heart [49], and heart failure-associated fibrosis [50]. Furthermore, exosomes are involved in cell-cell communication processes, and previous studies have demonstrated that metastatic cell-secreted exosomes were rich in POSTN content, and regulated cancer metastasis [51]. Therefore, POSTN in CM-derived exosomes might function as a key downstream effector of Ang II, promoting fibrotic effects in cardiac tissue. DCN, a small leucine-rich proteoglycan, is a pivotal effector with anti-fibrotic properties, that suppressed cardiac fibrosis by modulating TGF-β/Smad and p38 MAPK signaling pathways [52]. It has been previously reported that rosuvastatin-treated patients have increased secretions of DCN and biglycan, which bind to COL1 and COL3, thereby modifying collagen matrix assembly, and fibril packing [53]. Our present study showed that SIM-treated CM-derived exosomes contained abundant DCN levels, and the migration of HCF cells was significantly suppressed by recombinant DCN protein treatment. These data indicated that SIM attenuation of cardiac fibrosis progression, mediated by CM-derived exosomes, might occur via targeting DCN expression.

Previous studies demonstrated that Ang II promoted fibrosis by inducing HCFs proliferation, differentiation, production, secretion of extracellular matrix proteins, and expression of pro-fibrotic genes, but the key microenvironmental events responsible for regulating HCFs and CMs remain unknown [3,17]. HCFs constitute the functional cellular components of the adult cardiac interstitium, and are enmeshed in the endomysial interstitial matrix that surrounds CM, which accounts for approximately 20% of the myocardial volume [54]. Fibroblast to myofibroblast transformation is an indispensable process in cardiac fibrosis [48]. Myofibroblasts highly express α-SMA, and secrete large amounts of collagen (e.g., COL1A1) and procollagen-associated proteins (e.g., LOXL2) in the cardiac interstitium [55]. In addition, recent studies have shown that various pro-fibrotic regulators, including TGF-β1, extra domain A-fibronectin, microRNAs, POSTN, β-catenin, and exosomes, regulate fibroblast to myofibroblast transformation [56].

In our study, to identify the inhibitory effects of SIM in myofibroblast transformation, collagen-associated protein expressions and collagen deposition in perivascular and cardiac interstitial tissues were measured. Our investigation showed that SIM suppressed both in vitro and in vivo myofibroblast phenotypic transformation, and collagen-associated protein expressions. Furthermore, statins have been reported to reduce fibroblast proliferation and migration after myocardial infarction [57]. Our data confirmed that SIM significantly inhibited fibroblast migration after Ang II exposure. Taken together, our findings suggested that SIM is a key regulator of CM-derived exosome secretion.

Our study has certain limitations. Firstly, we did not explore clinical correlations between SIM and cardiac fibrosis; therefore, the results of this study should be extrapolated to humans with caution. Secondly, the exosome isolation was only from the human CM culture medium, which restricts their applicability to in vitro systems; exosomes should also be collected from cardiac fibrosis animal models in future experiments. Thirdly, although previous studies indicated that SIM has similar mechanisms and functions with other statins (ex. atorvastatin) [25,58], the effect of other statins in regulating exosome-dependent CF activation in Ang II-induced cardiac fibrosis remains uncertain. Fourthly, previous studies have shown that HCF-derived exosomes regulate cardiac repair, cardiac remodeling and CM hypertrophy in cardiac disease [41,43]. However, in this study, we did not have sufficient resources to explore the regulatory effects of SIM in fibroblast-derived exosomes that are associated with cardiac fibrosis. It has also been reported that microRNAs in secreted exosomes play a key role in the regulation of cardiac fibrosis pathology [44]. Thus, further studies should be performed to explore the in vitro and in vivo effects of SIM in exosomal microRNA and POSTN expressions.

## 5. Conclusions

**Figure 8:**
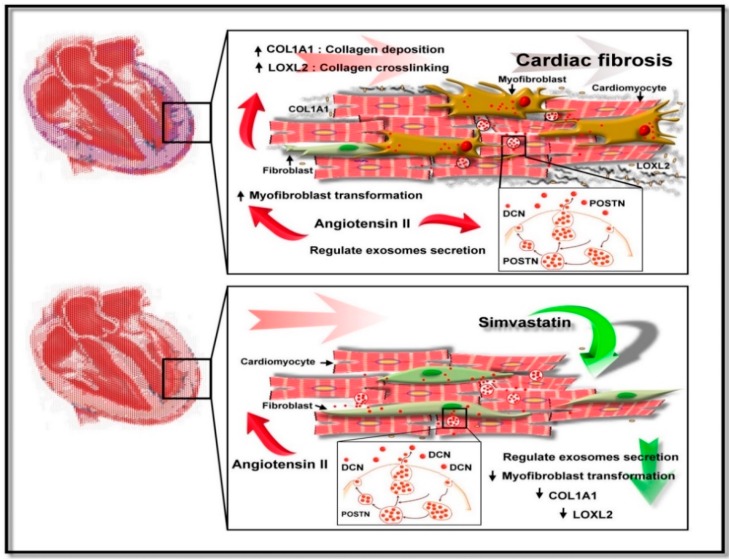
Suggested mechanism underlying Simvastatin-induced suppression of Ang II-mediated fibroblast. SIM might suppress fibroblast to myofibroblast transformation, collagen production/deposition, and increased fibroblast motility by inducing cardiomyocyte secretion of exosomal proteins.

We characterized SIM as a potential inhibitor of cardiac fibrosis mediated by CM-derived exosomes and showed that SIM might regulate fibroblast to myofibroblast transformation, collagen deposition, and fibroblast migration. SIM might also increase the expression of the exosomal anti-fibrotic protein DCN, while inhibiting expression of the Ang II-induced exosomal pro-fibrotic protein POSTN, to attenuate CM-derived exosome-mediated cardiac fibrosis. Our proposed model of the SIM effect on cardiac fibrosis is provided in Figure 8. Because SIM attenuated cardiac fibrosis progression via regulation of cell-cell communication within the myocardial microenvironment, it could serve as a therapy to reduce the progression of cardiac fibrosis. Further studies of SIM-regulated signaling pathways, which are implicated in exosome secretion and transformation from fibroblast to myofibroblast, are required to validate the therapeutic potential of SIM in cardiac fibrosis.

## Supplementary Materials

The following are available online at https://www.mdpi.com/2077-0383/8/6/794/s1, Figure S1: Simvastatin suppresses (Ang) II-mediated collagen deposition in vivo.
