# Supplementary material for: Simvastatin Attenuates Cardiac Fibrosis via Regulation of Cardiomyocyte-Derived Exosome Secretion
Source: J Clin Med. 2019 Jun 4;8(6):794. doi: 10.3390/jcm8060794 (PMC6617127; doi:10.3390/jcm8060794)
Supplement: Supplementary file 1 [file jcm-08-00794-s001.pdf]

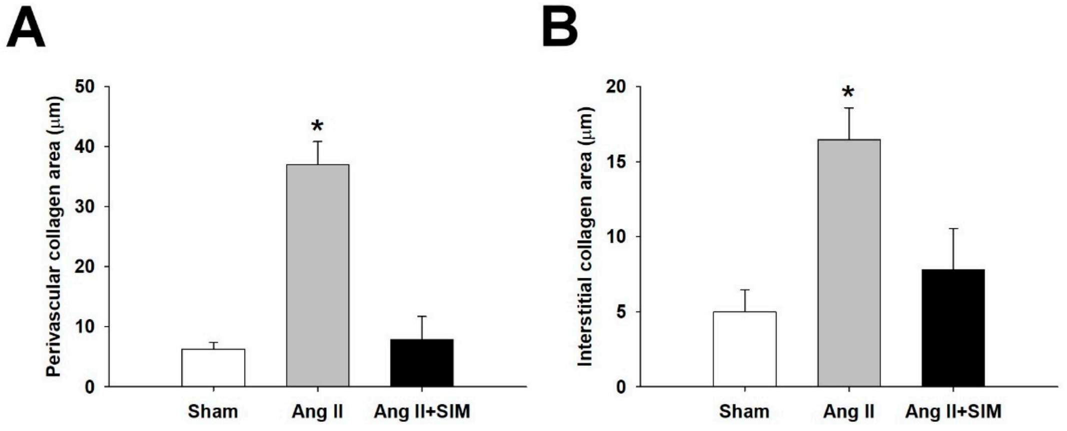

Supplemental Figure 1. Simvastatin suppresses (Ang) II-mediated collagen deposition in vivo. Male Sprague-Dawley rats were treated with (Ang II, 1 mg/kg/day) or Ang II + simvastatin (SIM, oral, 10 mg/kg) for 28 days. Perivascular and interstitial collagen fibers expression were determined and quantified by transmission electron microscopy (TEM) analysis (n = 3). For all comparisons, **p* < 0.05 vs. sham.
